# Improving Body Mass Index in Students with Excess Weight through a Physical Activity Programme

**DOI:** 10.3390/children9111638

**Published:** 2022-10-27

**Authors:** Mădălina Doiniţa Scurt, Lorand Balint, Raluca Mijaică

**Affiliations:** 1 Sports Science and Physical Education, IOSUD—Transilvania University of Brasov, Resident Physician-Specialization: Radiology and Medical Imaging, Hospital CLINICO Valencia, 46010 Valencia, Spain; 2 Department of Physical Education and Special Motricity, Faculty of Physical Education and Mountain Sports, Transilvania University of Brasov, 500036 Brasov, Romania

**Keywords:** physical activity, programme, children and adolescents, excess weight, urban environment

## Abstract

The obesity epidemic among young people can be tackled through regular physical activity. For this purpose, we developed and implemented a physical activity programme (PAP) that we carried out in students’ free time during the school year 2018–2019. The target group consisted of 79 students with excess weight, aged between 12 and 15 years, selected from an initial sample of 495 students from 5 pre-university education units located in an urban area. That group followed a differentiated PAP for 26 weeks. The impact of the programme highlighted the following points: the average physical activity/week for the entire sample of subjects was 3.67 physical activities, with an allocated time/week ranging from 1 h 30 min to 3 h; in terms of effort intensity, 7.70% of the activities were performed at low intensity, 75.07% at medium intensity and 17.23% at submaximal intensity. At the end of the programme, out of 79 subjects who were overweight/obese at the initial testing, 37 improved their body composition at the final testing, with a healthy BMI. It was also found that there is a negative correlation coefficient (r = −0.23) between the time spent performing physical activities and the BMI of the subjects.

## 1. Introduction

Physical activity habits have changed over the years, and today, increasing numbers of young people are tending to become less active from a motor perspective, a fact that is reflected in their lifestyles. The reality that current research reveals is that the evolution of physical activity has stagnated in recent decades among all categories of citizens, and the percentage of those who practiced but have stopped has increased alarmingly [1]. A study on current trends in physical inactivity in adolescents was published at the end of 2019 [2]. This study pointed out that in 2016, overall, 81% of the respondents (adolescents aged 11–17 years) were not physically active enough (77.60% of boys and 84.70% of girls), and that by territorial socioeconomic level, the prevalence of physical inactivity in 2016 was 84.90% in underdeveloped countries and 79.40% in economically developed countries. 

The reality of the situation, particularly in adolescence, applies to virtually any other citizen, regardless of age or country of origin. About all of the above, by a relatively simple differentiation, it can be said that some are more physically active, and others are less likely to participate or to devote time consistently and significantly to this kind of biological effort (physical, mental, emotional). Explanations along this line of selective human behaviour are quite complex as the behaviours are multifactorially determined and, to simplify things, most researchers, rightly perhaps, choose one or another of the possible and relevant variables that determine physical activity or inactivity as the topic of study. Therefore, there are studies that generally address the following variables: age [3,4,5,6,7,8,9,10,11], gender identity [1,10,12,13,14,15,16,17,18,19,20,21,22], socioeconomic and education level [23,24,25,26,27,28,29,30,31], personality characteristics/motivation [12,15,32], social environment/family and friends [12,26,31,33], as well as environmental setting [4,34,35,36], all of which can influence the level of physical activity as a means of maintaining and/or improving health. Some of the variables listed above are not changeable (such as age, gender), but the others can be altered to a certain extent (level of education, subjects’ motivations, social and proximal environment in which each individual lives). 

Closely related to health, as a direct cause–effect relationship, is the concept of lifestyle [37,38], presented as a subjective term, encompassing different aspects. One of these is physical activity, which if performed with an appropriate frequency, intensity and duration, is an integrated factor in the so-called healthy lifestyle, which decisively contributes to the maintenance of health and quality of life [39]. In the same vein, any physical exercise performed sporadically does not become part of a stable lifestyle, and its beneficial influences cannot make their presence known.

Paradoxically perhaps, in this area of interest are taken as subjects of studies, to a large extent, only young people, namely “normal” people, and those who practice should be in the forefront of research attention; under these circumstances, it would be justified to be young people/adolescents who for one reason or another have deviated from the state of normality. Our study is also part of this category, which aims to support, in a proactive manner, adolescents with excess weight (of nonendocrine nature), considering that they are the first to either self-exclude or are excluded by others from the enjoyment of physical activities and as a result are not the beneficiaries of this kind of habit, becoming, in the not too distant future, those who will suffer from serious health conditions such as: cardiovascular diseases, diabetes, immune deficit liver diseases.

Specialized studies approach the category of overweight subjects, in most cases, through the prism of the subjects’ physical activity/inactivity and only through questionnaires. These are more of an observational nature, being “passive” studies that only point out the problem in question. For example, there are international studies showing an inverse relationship between physical activity and body fat [40,41,42] and the degree to which physical inactivity contributes/predisposes to increased levels of overweight among the infantile population compared with those who declare themselves physically active [43]. Along the same lines, other studies using objective methods to assess physical activity levels have shown negative associations between physical activity and the development of excess weight, obesity and central/abdominal adipose tissue among children and adolescents [44,45,46]. Similarly, in a cross-sectional study conducted with adolescent schoolchildren [47], an inverse relationship was found between physical activity and body weight. The results indicated that those who suffered from overweight and obesity spent less time engaged in physical activity than adolescents with a normal body weight. In the same observational manner, a Norwegian study [48] revealed that a substantial decrease in the level of physical activity among adolescents led to a significant increase in body mass index over the same period. Based on this argument, the authors of the study emphasize the importance of maintaining physical activity levels in order to reduce negative trends in body mass index growth. In Romania, research in this area has been largely of a constative nature and, moreover, without any connection with physical activity, presenting only statistical data either from interested institutions or researchers in fields related to physical education and school sport. For example, in an urban study conducted in 2008 on a group of 7904 children and adolescents in grades I-XII from 20 schools and high schools, prevalences were shown of overweight of 12.84% and obesity of 8.29%. Adolescents in this study had lower-than-average prevalences of, respectively, 7.66% for overweight and 3.81% for obesity [49]. In Timiș County (located in western Romania), according to another study [50] conducted between 2010 and 2011 on a total of 3731 subjects aged 7 to 19 years, the overall prevalence of overweight was 18.20% (boys, 20.70% and girls, 16.30%), and that of obesity was 7.20% (boys, 9% and girls, 5.80%). Overweight was more common in the urban areas, a percentage of 18.20%, compared with 17.90% in the rural area. Another observational study carried out over a period of 10 years (2006–2015) on 25,060 subjects aged between 6 and 19 years coming from 8 counties in Romania [51] showed that 28.30% of them had excess weight (obesity and overweight). The same study also finds that the prevalence of excess weight in children and adolescents in urban areas (29.5% = 18.30% overweight and 11.20% obesity; *n* = 20,137 subjects) is higher than that of children and adolescents in rural areas (22.90% = 14% overweight + 9.90% obesity; *n* = 4923 subjects). The examples are endless, but the important point is that in Romania, there is still little emphasis on the health-generating value of physical exercise practiced in one form or another continuously throughout life.

All this research, as well as other studies not mentioned here, is indeed a wake-up call for responsible entities (institutional and nongovernmental alike), who must take appropriate measures to prevent this globalized phenomenon, currently called the obesity epidemic. For this goal, however, proactive actions/practices are needed, not only to identify but, importantly, also to mobilize young people with excess weight in the constant practice of physical activities, especially in their free time, following a proper methodology. This is also the major goal of our study, which aimed to design and implement a physical activity programme (PAP) exclusively for adolescents aged 12 to 15 years with excess weight (overweight or obesity). Our working hypothesis was that the body mass index in adolescents with excess weight would correlate with the total amount of physical activity that the subjects performed in their free time in completing an average-duration physical activity program.

Following the implementation of this programme, some results were obtained that we consider encouraging. They offer real/objective perspectives on improving the body mass index for the study subjects according to their proactive attitudes toward these kinds of activities. The data highlighted for each subject how much time they dedicated to physical activities (sequentially averaged/day/week/month and overall for the 6 months) and what type of activities they enjoyed, as well as with what effort intensity they chose to perform their preferred activities. These data were finally correlated with the body mass index to demonstrate the existence or nonexistence of relationships there between. 

## 2. Materials and Methods

### 2.1. Methodology of the Physical Activity Programme

The physical activity programme (PAP) was created at our initiative as an operational model that could be disseminated among students with excess weight according to BMI and benefited, for the implementation of its contents, from the guidance of several specialists and volunteers in the field of sports science and physical education from the Transilvania University of Brasov Faculty of Physical Education and Mountain Sports. In order to be able to cover the entire range of activities related to our study, starting with the initial and final anthropometric measurements, the presentation and implementation of the PAP programme, as well as the monitoring/evidence and centralization of activities, we turned to the help of 5 physical education teachers from the subjects’ schools of origin, as well as to a university professor (teacher), and last but not least, to 5 students from our institution, from the study program Kinetic Therapy and Special Motricity.

In addition, we followed the recommendations of the World Health Organization (WHO) regarding the characteristics of our target group (young people with excess weight), namely at least 60 min/day of moderate to vigorous physical activity. When designing the PAP, it was also taken into account the idea that physical activity should be mostly aerobic but include other types of activities that contribute to the strengthening of the musculoskeletal system [52].

To select the subjects for our research, we performed anthropometric measurements on a larger group of students (Section 2.3) from among whom we extracted those with excess weight. Specifically, we measured height with a thaliometer and weight with an electronic scale, respecting the recommended methodologies in both measurement procedures, including regarding the summary clothing of subjects and lack of footwear. We next used these measurements to calculate the body mass index (BMI) of each subject (according to the formula: BMI = weight/height^2^), thus obtaining the sample of subjects targeted in our approach.

In developing the methodological guidelines of the PAP, we started from the assumption/recommendation that, in general, children and adolescents (6–17 years) with excess weight and/or who are usually physically inactive may not be able to practice 60 min of moderate (according to WHO) let alone increased physical activity. In this context and for our subjects, we felt that they should start in the PAP with moderate intensity physical activity and gradually increase the frequency and time allocated to physical activity to reach the target of 60 min of physical activity/day after a reasonable period of time. Therefore, it was suggested that for the activities carried out with higher intensity, the students should progressively incorporate them into their individual activity plans until they can perform high-intensity activities 2–3 times/week. 

In the model PAP, which was designed for 26 weeks and distributed to students to guide their choices, 5 weekly sessions of physical activity were proposed, with an intensity generally between 60 and 70% of the maximum heart rate/subject. During these sessions, we suggested for the weekend physical activities carried out in nature (hiking, cycling, sports orientation, etc.), all with a marked aerobic energy substrate and usually recommended for subjects with excess weight.

Additionally through the designed programme, we wanted to ensure that physical activity/exercise was not abandoned during the study, thus combating the sedentary lifestyle so common today at these ages. At the same time, the programme was developed in such a way as to allow the participants to modify its contents according to their personal preferences.

The physical activity volume, in terms of frequency, the intensity of physical effort and the duration and type of physical activity chosen, was monitored through the physical activity journals that we designed and distributed to the students. Periodically, from these journals, the different quantitative indicators needed for the processing of the executive/practical components of the study were extracted. The students recorded their frequency of physical activities, duration and activity type after each execution in tables in their journals.

In the case of identifying the zones of physical effort (low, aerobic 1, moderate, aerobic 2 and submaximal, aerobic–anaerobic), recorded in the same format of the journal, the subjects were asked to self-determine their heart rate (either by measuring the heart rate by palpating it at the level of the radial artery or by downloading the Heart Rate Monitor-Pulse App from any type of smartphone from the Play Store menu) at 6–8 min after starting an activity, as well as at 15, 20 and/or 30 min (depending on the type of activity), finally averaging the 2 or 3 values obtained. As indicative values for framing the effort intensity zones for ages 12–15 years, we chose the intervals: 130–150 rpm (low); 155–170 rpm (moderate); >175 (submaximal).

Due to the variety, attractiveness and fun that the physical activities of PAP can generate, we encouraged family and friends, even if they were not overweight, to participate together with the subjects to support, encourage and motivate them in their efforts to complete their activities.

Regarding the locations for practicing physical activities that make up the PAP (these being extracurricular activities), the following were suggested to students/their parents: spaces in one’s own school, neighbourhood or home, a park or any other accessible public space that offers physical safety and has a purpose that allows the practice of these types of activities. 

All this information, as well as the benefits of completing the PAP, were presented at the beginning of the school year (September 2018, after the target group was created), within the pre-university education units involved in the study: physical education teachers, students and their parents, from which the acceptance agreement was obtained. As a result, the study was conducted in accordance with the Declaration of Helsinki and approved by the Institutional Examination Board of the Faculty of Physical Education and Mountain Sports of the Transilvania University of Brasov (No. 243 from 26 September 2018), and informal consent was obtained from all subjects (the parents of the 79 students) involved in the study.

As a final clarification regarding the methodological aspects of the application of the PAP, we would like to mention that all the proposed activities were chosen in such a way as to have a non-competitive character, thus encouraging the development of confidence in one’s own strengths, without favouring the manifestation of inferiority complexes and with the contents adapted to individual motor/physical capabilities. 

### 2.2. Presentation of the Contents and Structure of the Physical Activity Programme (PAP) for Schoolchildren with Excess Weight

The PAP was designed on 4 modules of physical activities: aerobic endurance; strength and muscular endurance; flexibility and weekend activities. Please note that these modules were not randomly selected. Their development was based on the recommendations of several studies carried out internationally that indicate that these types of activities are the most effective for adolescents. It is considered that from a physiological point of view, children and adolescents (6–17 years) adapt easily to physical activities practiced to develop muscle strength and endurance [53,54], aerobic endurance [55,56] and flexibility/mobility [57,58]. However, because the musculoskeletal system is still immature at this age, children and adolescents should not engage in excessive amounts of high-intensity physical activity.

Within each programme out of the 4 proposed, for each physical activity, we estimated the total caloric consumption generated by its execution. These values were obtained experimentally by measuring the MET (the metabolic equivalent of a task/activity) in an obese 13-year-old girl weighing 70 kg and an overweight 15-year-old boy weighing 67 kg. Please note that MET is the unit of measurement for oxygen consumption, and for calculating the total calories consumed/physical activity, the oxygen consumption and the body weight were used, in addition to other parameters such as age, stature and total time dedicated to physical activity. The calculator used to obtain these data is available online at https://healthyeater.com/calories-burned (accessed on 3 October 2018). Regarding the weekend activities module, in order to estimate the caloric burns occurring in the body, we took into account constants such as: body weight, distance covered (in kilometres), type of terrain (presence of uphill and/or downhill sequences) and level difference. The calculator used is available online at: https://caloriesburnedhq.com/calories-burned-hiking/ (accessed on 5 October 2018).

Table 1 present the main indicators of each module.

These modules of the PAP represent diversified offers for physical effort. Due to this fact, they are not of interest in terms of their contents (which are obviously different) but more for the relevant indicators they develop, such as: the number of activities carried out/subject, their average duration/week and the intensity zones in which they fall.

As a way of presenting the PAP document to the students, physical education teachers and the students’ parents, each module included: preamble, objectives, application requirements/recommendations and methodological conditions (as appropriate) and examples of activities in different intensity steps with the equivalent in caloric burn. 

From a longitudinal perspective, the model PAP, designed over an effective duration of 6 months (26 weeks effectively), was divided into 4 sections with different steps/purposes of action as follows: weeks 1 to 4 (the settling-in period); weeks 5 to 8 (the development period); weeks 9 to 12 (the optimization period); weeks 13 to 26 (the completion period).

Each physical activity session (regardless of the module chosen) consisted of 3 parts that were recommended to students, namely:

1. A body preparation phase (warm-up) lasting 5–10 min, consisting of 4–5 min of brisk walking/running/cycling, etc., followed by dynamic joint mobility exercises, 5–6 min.
2. A main part, lasting 20–30 min, in which exercises on a suitable energy substrate/activity are combined to develop fitness (depending on the module chosen).
3. A body recovery phase after exercise (winding down), lasting 5–10 min, which is important for lowering the heart and breathing rates and is achieved by performing a active physical mobility exercises (stretching), static and/or dynamic, to avoid muscle pain.

### 2.3. Research Subjects, Inclusion/Exclusion Criteria

The experiment was conducted from September 2018 to June 2019. The target group for the study consisted of 79 subjects with excess weight (38 girls and 41 boys) from General Schools No. 11, No. 13, No. 15, No. 19 and No. 30 of the Municipality of Brasov, aged between 12 and 15 years. In the 5 educational units, we had access through the collaboration protocol concluded between our academic institution and the Brașov County School Inspectorate (partnership agreement no. 298/18 October 2017). The selection of subjects was targeted on multilayered sampling to cover a varied population in terms of age and gender. From an initial group of 494 pupils, the eligible population was selected: 79 subjects with excess weight (52 overweight subjects, group SP, and 27 obese subjects, group O). We divided the final study sample into 4 subgroups for analysis and comparison: obese girls; obese boys; overweight girls; overweight boys).

- Inclusion criteria: pupils from secondary school (grades V–VIII); obesity: BMI ≥ 95th percentile (+2SD) for gender and age; overweight: 85th percentile ≤ BMI < 95th percentile (+2SD) for gender and age; possibility of monitoring and evaluating the results by completing the proposed physical activity programme.
- Exclusion criteria: impossibility to monitor subjects; refusal of parents and/or students to participate in the study.

### 2.4. Statistical Methods Used in Research

The processing and interpretation of the collected data was carried out using the Statistical Package for the Social Sciences Program (SPSS version 25.0; IBM Corp., Armonk, NY, USA).

## 3. Results

### 3.1. Identification of Subjects with Excess Weight at the Initial Measurement 

Table 2 and Table 3 present the distributions of the students’ height and weight at the initial measurement, respectively. 

Table 3 indicates that most of the subjects had a heathy body weight and only about one-third had a very high body weight. However, calculating the body mass index showed that all subjects had excess weight (Table 4).

The subjects’ distribution by grade is as follows: 15 students (18.99%) were in the 5th grade, 16 students (20.25%) in the 6th grade, 23 students (29.11%) in the 7th grade and 25 students (31.65%) in the 8th grade. In terms of age, the oldest students were the 8th-grade students, with an average age of 15 years; followed by the 7th-grade students, with an average age of 14 years; 6th grade, average age 13 years; and 5th grade, average age, 12 years. 

At the end the physical activities programme (April 2019), we collected a significant amount of data in the following areas: volume of activity performed/subject, expressed as the number of activities performed; time allocated to physical activities (expressed in minutes and hours); the intensity of the physical activities; and the subjects’ actual choices of the activities provided in the PAP. Below, we present the findings according to the four student subgroups: overweight boys and girls and obese boys and girls.

### 3.2. Average Volume/Frequency of Physical Activities Performed According to the Body Mass Index and Gender of Subjects, Post-Impact PAP

Over the 26 weeks, overall, the subjects in our study (the 79 subjects—38 girls and 41 boys) performed an average of 95.36 physical activities/subject, the fewest being 64 and the most, 124 (Table 5), for an average for the entire sample of subjects of 3.67 physical activities/week. This result was obtained by dividing the average physical activity/subject (95.36) by the duration of the PAP (26 weeks).

It can also be observed that based on the body mass index, over the entire duration of the programme, obese girls performed more activities on average than obese boys, a fact found also in the case of overweight girls in relation to overweight boys (Table 5). 

**Figure 1:**
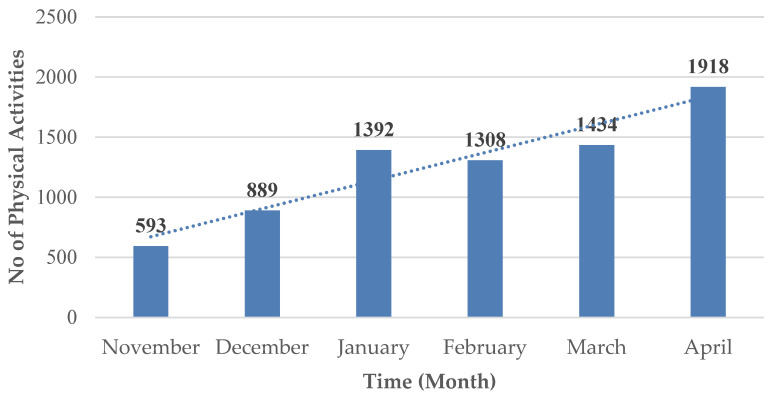
Distribution of the total number of physical activities /month.

Both overweight boys and girls performed more physical activities than the obese girls and boys by an average difference of 7.36 activities. Over the 6 months of the programme, the 79 students performed a total of 7534 activities, an overall average of 3.67 activities/week/subject. Figure 1 presents their activities by month. It can be observed that most physical activities were carried out in April, while the fewest were carried out in November.

### 3.3. Averages/Subgroups of the Time Allocated to the PAP Sessions

**Figure 2:**
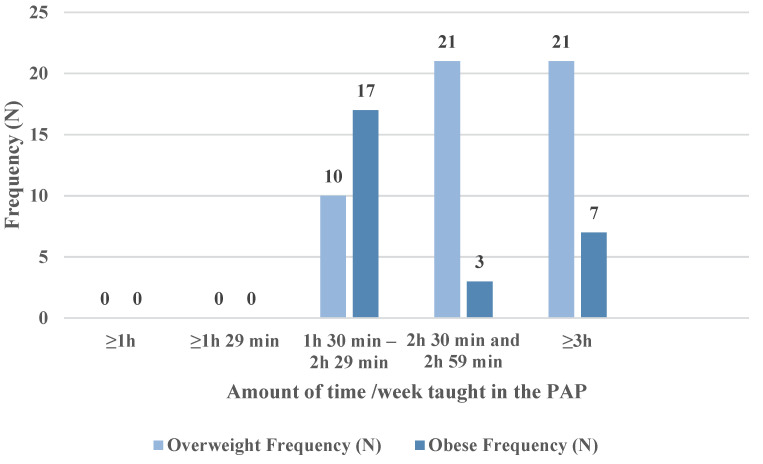
Distribution of total weekly time spent on physical activity.

The total amount of time dedicated to the PAP was calculated for the entire period of the programme (1 November 2018–30 April 2019), according to the body mass index and gender of the subjects concerned. On an overall level (79 subjects), the time dedicated to the physical activity programme shows an increasing trend from month to month: from a total of 916 h 8 min in November 2018 to a total of 1101 h 10 min in April 2019. In obese subjects, girls spent more time on physical activity (983 h 24 min) than boys (883 h 57 min). In the case of overweight subjects, girls (1990 h 29 min) and boys (1976 h 23 min), allocated approximately the same total amount of time to the program. Table 6 presents average times spent on the physical activities according to weight group (obese or overweight) and time interval (between 1 h 30 min–2 h 29 min and ≥3 h), as well as their number/activity time interval/week (Table 6 and Figure 2). 

### 3.4. Averages/Subgroup Effort Intensity of the Physical Activities 

To determine the average of the physical activities/subject according to the intensity of the physical effort, we cumulated the frequency of the physical activities performed (percentages) by each subject, and subsequently, divided the obtained result by the total number of subjects corresponding to each of the 4 subgroups. 

**Figure 3:**
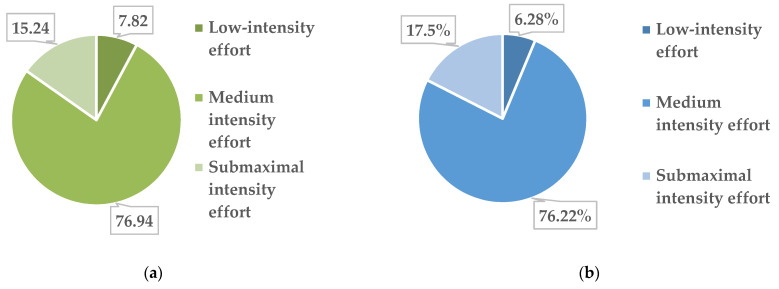
Distribution of physical activity by intensity of the physical effort in obese girls (**a**) and overweight girls (**b**).

The collected data highlight the fact that during the 26 weeks of activity, in the group of obese girls (13 girls), out of the total of 1253 physical activities performed, 98 were performed with a low intensity of physical effort, 964 were performed with an average intensity and 191 were at submaximal intensity. The percentages physical activities/effort zones for obese girls are shown in Figure 3a.

In overweight girls, out of the total of 25 subjects, we found that out of the total of 2515 physical activities carried out, 158 were performed with a low intensity of physical effort, 1917 with an average intensity and 440 at submaximal intensity. In Figure 3b, these data are presented as percentages.

**Figure 4:**
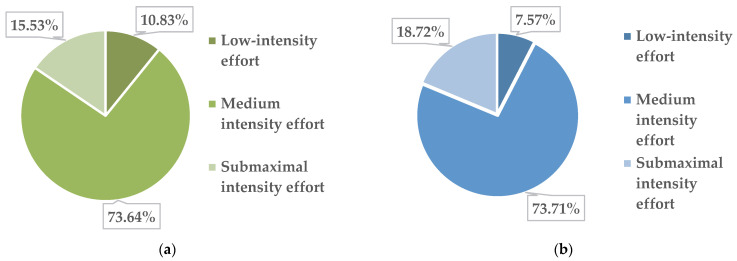
Distribution of physical activity by intensity of the physical effort in obese boys (**a**) and overweight boys (**b**).

In the subgroup of obese boys (14 subjects), out of the total of 1191 physical activities, 129 were performed with a low intensity of physical effort, 886 with an average intensity and 176 with a submaximal intensity. The corresponding percentages are presented in Figure 4a.

In overweight boys, out of the total of 27 subjects, out of the total of 2575 physical activities, 195 were performed with a low intensity of physical effort, 1898 physical activities with an average intensity and 482 at submaximal intensity. The corresponding percentages can be found in Figure 4b. 

Table 7 presents the 52 overweight and 27 obese (at initial measurement) study subjects’ numbers of physical activities (AF), their percentages (%) and the intensity zones of the activities (A, B, C).

### 3.5. Types of Activities Chosen by the Study Subjects

**Figure 5:**
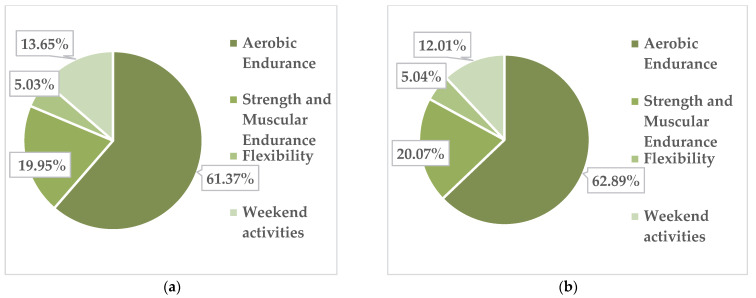
Frequency of preferred activities/module in the physical activity programme in obese girls (**a**), obese boys (**b**).

**Figure 6:**
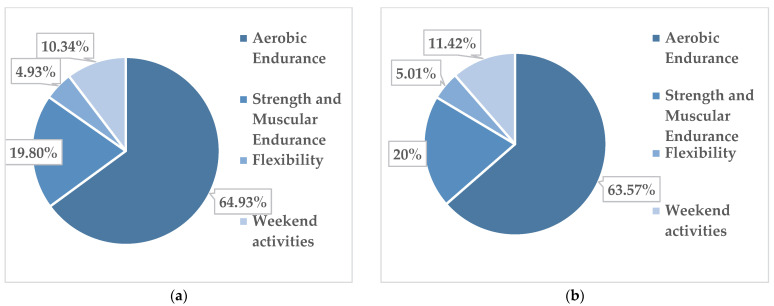
Frequency of preferred activities/module in the physical activity programme in overweight girls (**a**), overweight boys (**b**).

Figure 5a,b and Figure 6a,b present the percentages of participants in each activity module for the obese and overweight girls and boys, respectively, for all 79 subjects. 

The data reflect the fact that all students, regardless of their subgroup, liked the physical activity modules in the following order: 1. aerobic endurance; 2. strength and muscular endurance; 3. flexibility; 4. weekend activities.

### 3.6. Interpretation of Final Results

At the end of the 6-month programme, we again measured the students’ height and weight and again calculated body mass index for the subjects in the four subgroups in order to ascertain if there are, especially in the latter parameter (BMI), significant changes resulting from the effect of the continued practice of physical exercise.

- In the final testing, the majority of the subjects measured, namely 75 subjects, had a normal stature index (Table 8).
- In the final measurement of weight, the students showed an average body weight of 66.57 kg, and the results deviate from the average by 12.13 kg. The majority of the subjects measured (77 subjects) had a normal body weight, and only 2 subjects still had a high body weight relative to age (Table 9).

**Table 8:** Distribution of height in the target group–final testing.

Statural Classification		Frequency	Percentage	Percentage of Measured Subjects	Cumulative Percentage
Height (cm)	Hyperstatural	1	1.30	1.30	1.30
	Normostatural	75	94.90	94.90	96.20
	Hypostatural	3	3.80	3.80	100
	Total	79	100	100	

**Table 9:** Distribution of weight in the target group–final testing.

Body Weight Classification		Frequency	Percentage	Percentage of Measured Subjects	Cumulative Percentage
Weight (kg)	Extra Heavy Weight	2	2.50	2.50	2.50
	Normal weight	77	97.50	97.50	100
	Very low weight	0	0.00	0.00	
	Total	79	100	100	

There were no significant differences between boys and girls in either height or weight at both the initial and final testings. The majority of the subjects out of the total of 79 (38 girls and 41 boys) were of normal height and of weight within normal limits.

- The analysis of the body mass index at final testing reveals the BMI at the time of final testing by gender. Basically, in order to centralize the results, we defined the categories of subjects according to the new body mass indices obtained after participating in the PAP. Out of the total of 38 girls identified at the initial testing as having excess weight (25 overweight girls and 13 obese girls; see Table 4), at the final testing (Table 10) after completing the PAP, 23 girls acquired a healthy BMI, 9 girls were classified as overweight and 6 girls were obese. In boys, out of the total of 41, initially 27 overweight and 14 obese (Table 4) in the final testing (after completing the PAP), 14 boys reached a healthy BMI, 17 boys were classified as overweight and 10 boys as obese.

**Table 10:** Distribution of body mass indices at the final testing.

BMI Classification		Frequency	Percentage	Percentage of Evaluated Subjects	Cumulative Percentage
BMI (kg/m^2^)	healthy	37	46.80	46.80	46.80
	overweight	26	32.90	32.90	79.70
	obesity	16	20.30	20.30	100
	Total	79	100	100	

**Figure 7:**
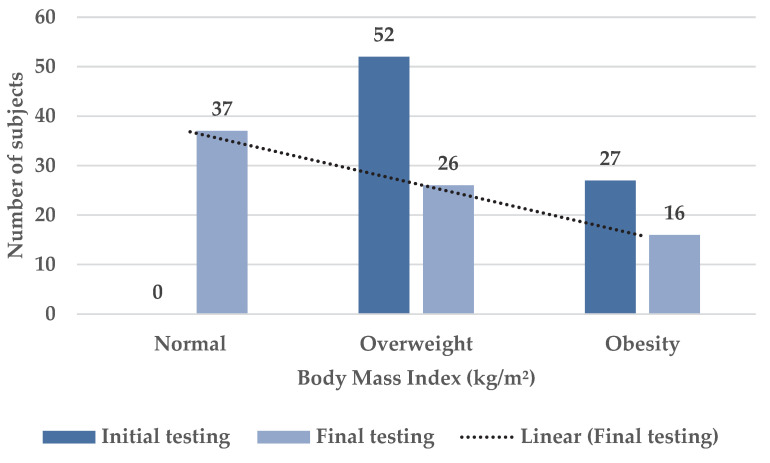
Body mass index distribution in initial and final testing for the target sample.

Regarding BMI, determined at the initial and final testings, it can be noted that at the initial test, 52 overweight subjects were recorded, and at the final test only 26 students remained classified as overweight. At the same time, of the 27 subjects identified with obesity at initial testing, only 16 subjects still showed obesity on final testing (Figure 7). 

**Figure 8:**
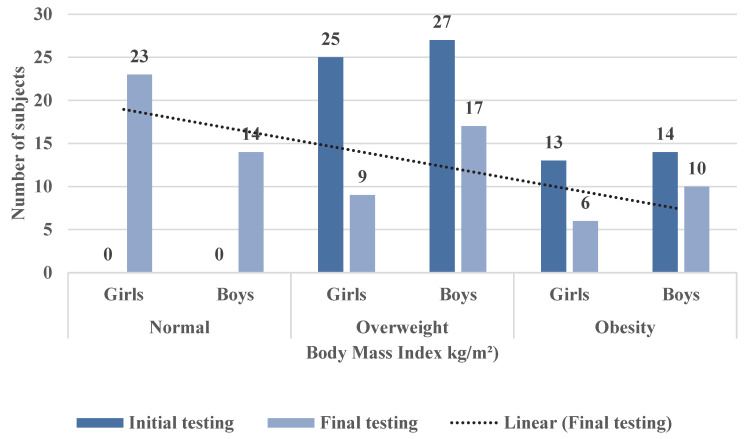
Distribution of initial and final body mass indices for the target sample by gender.

**Figure 9:**
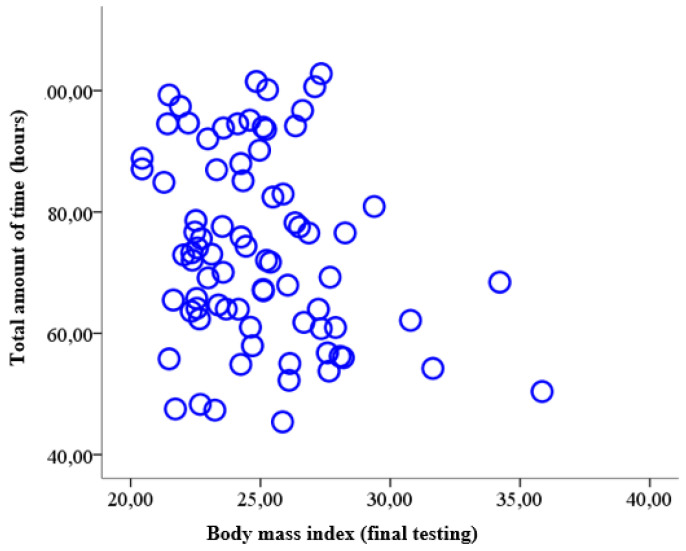
Scatter diagram for the relationship between the body mass index determined at the final testing and the total amount of time devoted to physical activity (hours).

With regard to body mass index by gender, as determined in the initial and final testings (Figure 8), it can be observed that:

- In the initial testing, 25 girls were identified as overweight, while in the final testing, only 9 girls were still overweight;
- In the case of overweight boys, in the initial testing, 27 boys were overweight, and in the final testing, 17 boys with overweight were identified;
- In the case of obese girls, 13 girls were recorded with obesity at the initial testing, and 6 girls were obese at the final testing;
- For obese boys, 14 were identified in the initial testing, while in the final testing, only 10 boys had obesity;
- Out of the total number of subjects (79), at the final testing, 37 subjects with a healthy body mass index were reported (23 girls and 14 boys).
- In concluding this study, we wanted to determine whether there was a relationship between body mass index (acquired at the time of final testing) and the total amount of time allocated by the subjects with excess weight to the physical activity programme (PAP). Thus, the materiality threshold (*p* = 0.04) obtained in the bivariate analysis between the BMI determined at the final testing on the one hand and the total amount of time devoted to PAP on the other hand shows that there is a statistically significant correlation. As we can see, this correlation is reversed (r = −0.23), which allows us to state that in the entire sample (79 subjects with overweight and obesity), a higher body mass index was associated with less time dedicated to the physical activity program (PAP) (Table 11 and Figure 9).

**Table 11:** Results of the Pearson correlation analysis between the body mass index determined at final testing and the total amount of time devoted to the physical activity programme (PAP).

		BMI-TF	VT. PAP
BMI-TF	Correlation coefficient	1	−0.23
	Materiality threshold (*p*)		0.04
	N	79	79
VT. PAP	Correlation coefficient	−0.23	1
	Materiality threshold (*p*)	0.04	
	df	79	79

The correlation is significant at a threshold of 0.05; BMI-TF = body mass index determined at final testing; VT. PAP = total amount of time dedicated to the physical activity programme.

Furthermore, the statistical analysis using the chi-squared test shows that there is a significant difference in the sample selected to participate in the PAP based on body mass index in terms of the total amount of time spent weekly on physical activities (χ^2^(9) = 19.33, *p* = 0.02). Along the same lines, the analysis of the data with the help of Fisher’s exact test leads us to the conclusion that there is a significant difference (Fischer’s exact probability *p* ≤ 0.001) in the total amount of time spent per week on physical activity between obese subjects (27 subjects, 13 girls and 14 boys) and overweight subjects (52 subjects, 25 girls and 27 boys) (Table 12). In other words, our study shows that overweight subjects (girls and boys) devoted more time to physical activity than obese students. 

The data presented confirm, from a statistical and mathematical point of view, our working hypothesis, which we mentioned in the introductory part of the study. 

## 4. Discussion

In Romania, the National Center for Evaluation and Promotion reported on the state of health (CNEPSS) for the 2016–2017 school year (study published in 2018), centralizing data regarding the prevalence of chronic diseases found in preschoolers and students, and highlighted the fact that in the second place is obesity of nonendocrine causes in both urban and rural areas [59]. For the urban environment, the following values are presented: 66.95% of children and young people are healthy weight, 21.75% are overweight and 11.3% are obese. Analysing the preliminary sample from our research, the data show us that of the total number of subjects measured (*n* = 494), a percentage of 84.01% are healthy weight and 15.99% are overweight, of whom 10.54% are overweight and 5.46% obese. As can be seen, our sample falls into lower values of excess weight compared with the national averages for Romania. We believe that this difference is due to the geographic area of the city where the measurements were made. Brașov is one of the biggest mountain tourist towns in Romania, with a multi-ethnic population, where there is a certain culture of practicing physical activities. This fact can be an argument for the mentioned differences. On the other hand, also for Romania, the World Obesity Federation estimated in 2016 that 4% of girls and 9.20% of boys aged 10–19 overweight /obese, thus resulting in a sum of 13.2%. Comparing this value with that of our sample (15.99%), which fits into a narrower age range (12–15 years), we can consider that the two means are quite close. Also, the same source [60] estimates that in the year 2030, in Romania, approximately 14.30% of the population aged 10–19 will be obese, and our initial value, related to the excess weight of the subjects, was 15.99%. At the end of the PAP, this percentage dropped significantly, to 8.50% (42 subjects with excess weight out of *n* = 494), with a difference of almost half of the initial percentage, 7.49%. In this context, we can say that the independent variable through which we intervened and which we monitored was given only by the continuous practice of physical activities (through PAP), and this fact determined an improvement in the body mass index at the level of the majority of the targeted subjects.

Regarding the number/frequency of physical activities recommended for children and adolescents, it is true that the World Health Organization [52] recommends practicing at least 60 min/day of moderate to high intensity physical activity (vigorous). We did not succeed in reaching this level of participation with our subjects, our average of physical activities/week having a frequency of 3.66 (global value). However, the same source believes that currently there is no clear evidence of an association between physical activities and managing a healthy weight, and more research is needed to determine the level of association between them. Our study, based on the collected data, is also oriented towards this shortcoming, and the statistical correlation that emerged as a result of our calculations (r = −0.23) shows that the students in our interventions with higher body mass indices spent less time dedicated to the physical activity program (PAP). In this context, also in the direction of those indicated previously, we found through the obtained data that there was a statistically significant difference (*p* ≤ 0.001) in terms of the total time dedicated weekly to the PAP between obese and overweight subjects. In other words, our study shows that overweight subjects (girls and boys), who in general allocated a higher proportion of time to PAP activities (80.80% in the intervals of 2 h 30 min and 2 h 59 min or ≥3 h), than the obese (37%), achieved a more significant improvement in body mass index in relation to the obese, a fact that can be considered as representing an association between physical activity and a healthy weight.

Regarding the contents of the four modules within the PAP (aerobic endurance; strength and muscular endurance; flexibility; weekend activities, mostly aerobic 1), the final results of our research show that they were correctly chosen, being effective for the intended purpose, and confirm the correctness of the recommendations offered by some specialized studies that consider that children and adolescents (6–17 years) can easily adapt to physical activities practiced in order to develop strength and muscular endurance [53,54], aerobic endurance [55,56] and flexibility/mobility [57,58].

Regarding the intensity zones of effort that we agreed on for the PAP, they are also supported by the recommendations of extensive specialist studies [61]. Thus, in these informative documents, for children and adolescents between 5 and 17 years of age, not only physical activities of moderate intensity but also physical activities of high intensity carried out at least 3 times a week including muscle strength exercises are recommended. We, through the PAP, offered to adolescents the opportunity to perform activities (regardless of their content) with the possibility of performing them in three intensity zones (low, moderate and submaximal). Monitoring this aspect during the 26 weeks, we found that overall, the intensity zone where the most activities were performed was the moderate one (75.07% of the total PAP activities), followed by the submaximal one (17.23%) and the one with low intensity (7.70%). Compared with the previously mentioned recommendations, our study does not fully comply with the provision for high-intensity physical activities, but taking into account the fact that the 79 subjects were all with excess weight at the beginning of the intervention and the source reference is given for those of healthy weight, we still consider that the indication in question was respected to a certain extent, and it follows that in the future, one should insist on performing physical activities that require a more sustained effort.

One last aspect we would like to point out is adolescents’ physical activity according to gender. Summarized data [21] referring to 20 studies from different countries found that in children and adolescents, boys in all countries were more active than girls. Along the same lines, [12] it was found that girls practice less physical activity regardless of the age analysed (from 11 to 16 years). According to the authors, for a long time, the socialization process has caused sports inhibition among girls because sports have been labelled a “male activity”.

Compared with the mentioned considerations, through the data of our research, we did not notice these differences in attitude between the 38 girls and 41 boys (79 subjects) included in the PAP. Our claims are based on the cumulative data obtained on the number of physical activities performed within the PAP, throughout the monitoring period (26 weeks). Thus, from the total of 7534 physical activities completed, the boys performed 3766 activities and the girls 3768. This equality in the data is given, we believe, by the way we constantly promoted and motivated the subjects (especially through the five physical education teachers who centralized the weekly records of the activities) to practice physical activities in view of our common goal, to improve the excess weight. We believe that the most important factor to consider when it comes to changing physical activity behavior is motivation, which is the inner force that determines the completion of a task for which a commitment has been agreed on.

Our study, admittedly conducted on a more specific sample, namely monitored adolescents with a certain level of excess weight, showed, we believe, that there are no major differences between the levels of participation of girls and boys in PAP activities and even in the types of agreed-on/accessed modules according to the centralized self-reports. 

As a general educational aspect, we are also not sure whether it is a good idea, from a pedagogical perspective, to group students in physical education lessons as a constant organizational form of directed motor activity according to normal and overweight categories and compare the manifested attitudes. After all, all children and adolescents are ours (from a didactic perspective), and we are therefore bound to support them in their own training. Perhaps even based on this idea, we decided that the activities in our study should be carried out in the students’ free time and differentiated according to their interests and enjoyment. We believe that when those responsible (we refer primarily to physical education teachers) adopt a positive pedagogy in their training approach (whether formal or non-formal), encourage the students, motivate them, show persuasive power through relevant arguments formulated according to the student’s level of understanding, constantly highlight each student’s progress, support the student in their most difficult moments, keep in touch with the students’ families, adopt social learning techniques through movement, etc., the results and attitudes towards physical activities can be surprising. Thus, first in our minds, then in the minds of teachers, and from there through the education provided to all young people and their families, the ideas that discriminate between the two sexes with regard to the practice of physical exercise in any form will fade over time, and even more, the psychological boundary given by the criteria of body mass index will be eliminated, everything being redirected towards the real pleasure that movement can generate for all regardless of age, body weight and any other criteria of human segregation. 

## 5. Research Limitations

The present study is only one section of a larger research project that also includes dietary aspects of students with excess weight, as well as measuring the levels of physical fitness in this target group before and after the impact of the physical activity programme. Due to lack of space, we have not been able to synthesize all the data we have available. The study is also focused on a selective sample in terms of inclusion criteria and limited in terms of number of participants, which is why the data presented need to be confirmed by other more comprehensive studies from a quantitative point of view and possibly extend to the rural school population.

## Figures and Tables

**Table 1:** PAP program features.

PAP—Aerobic Endurance Module			
Session Frequency	Intensity	Dosing	Type of Activity
5–7 times/week	Moderate-high 130–150 heartbeats/min. 155–170 heartbeats/min.	15–60 min/session (With an upward dynamic in volume)	Running, skating, cycling, swimming, cross-country skiing or any other cyclical physical activity
PAP—Strength and Muscular Endurance module			
Session frequency	Intensity	Dosing	Type of activity
at least 3 times/week	Moderate 130–150 heartbeats/min.	8–10 repetitions; 8 to 10 exercises/set of exercises	A wide range of exercises for harmonious physical development, toning and muscle trophicity.
PAP—Flexibility module			
Session frequency	Intensity/Amplitude	Dosing	Type of activity
at least ≥3 times/week	There must be no pain	Dynamic exercises—Position must be held for 4–5 s, 3–5 repetitions Static exercises—Position must be held for 10–20 s.	Pilates stretching (Active and combined)
PAP—Weekend activities module			
Session frequency	Intensity	Dosing	Type of activity
1 time/week	Moderate 130–150 heartbeats/min.	Depending on the chosen route (2 to 4.2 km)	Hiking, Theme parks

**Table 2:** Distribution of height in the target group–initial testing.

Statural Classification		Frequency	Percentage	Percentage of Measured Subjects	Cumulative Percentage
Height (cm)	Hyperstatural	1	1.30	1.30	1.30
	Normostatural	74	93.70	93.70	94.90
	Hypostatural	4	5.10	5.10	100
	Total	79	100	100	

**Table 3:** Distribution of weight in the target group–initial testing.

Body Weight Classification		Frequency	Percentage	Percentage of Measured Subjects	Cumulative Percentage
Weight (kg)	Extra Heavy Weight	23	29.10	29.10	29.10
	Healthy weight	56	70.90	70.90	100
	Very low weight	0	0.00	0.00	
	Total	79	100	100	

**Table 4:** Distribution of initial body mass indices by gender.

			BMI (kg/m^2^)		Total
			Overweight	Obesity	
Gender	Girls	Frequency	25	13	38
		Percentage (%)	65.80%	34.20%	100%
	Boys	Frequency	27	14	41
		Percentage (%)	65.90%	34.10%	100%
Total		Frequency	52	27	79
		Percentage (%)	65.80%	34.20%	100%

**Table 5:** Total physical activity by body mass index and gender.

BMI	Gender	N	Frequency Average	Standard Deviation	Average Standard Error	95% Conf. Range		Minimum	Maximum
						Min.	Max.		
O	Girls	13	96.38	21.02	5.83	83.68	109.09	73	123
	Boys	14	85.07	13.30	3.55	77.39	92.75	69	123
Total		27	90.72	18.04	3.47	83.38	97.66	69	123
SP	Girls	25	100.60	13.92	2.78	94.85	106.35	75	124
	Boys	27	95.37	18.87	3.63	87.91	102.83	64	124
Total		52	97.98	16.72	2.32	93.23	102.54	64	124

O = obesity; SP = overweight.

**Table 6:** Distribution of total weekly time spent on physical activity by weight group and time interval.

BMI (kg/m^2^) Quantitative Indicators			Average Time Volume/Week Dedicated to the Physical Activity Programme by Subject/Group					Total
			≥1 h	≥1 h 29 min	1 h 30 min–2 h 29 min	2 h 30 min and 2 h 59 min	≥3 h	
Groups	Overweight	Frequency (N)	0	0	10	21	21	52
		Percentage (%)	0.00%	0.00%	19.20%	40.40%	40.40%	100%
	Obese	Frequency (N)	0	0	17	3	7	27
		Percentage (%)	0.00%	0.00%	63.00%	11.10%	25.90%	100%
Total		Frequency (N)	0	0	27	24	28	79
		Percentage (%)	0.00%	0.00%	34.20%	30.40%	35.40%	100%

**Table 7:** Total number of performed physical activities by gender, weight group, and intensity of the physical effort.

BMI (kg/m^2^)	Gender	No. of Subjects	Freq. N/AF	Total		
				A	B	C
overweight	Girls	25	No. AF	158	1917	440
			%	6.28%	76.22%	17.50%
	Boys	27	No. AF	195	1898	482
			%	7.57%	73.71%	18.72%
Total		52	No. AF	353	3815	922
			%	6.94%	74.95%	18.11%
obesity	Girls	13	No. AF	98	964	191
			%	7.82%	76.94%	15.24%
	Boys	14	No. AF	129	877	185
			%	10.83%	73.64%	15.53%
Total		27	No. AF	227	1841	376
			%	9.29%	75.33%	15.38%
Total		79	No. AF	580	5656	1298
			%	7.70%	75.07%	17.23%

A = low intensity, B = medium intensity, C = submaximal intensity, AF = physical activity.

**Table 12:** Statistical significance of the total amount of time devoted weekly to the physical activity programme based on excess weight category and gender.

	Value	Degrees of Freedom	*p*
Pearson Chi-Square	16.01	2	0.001
Fisher’s Exact Test	15.53		0.001
Valid responses	79		

## Data Availability

Not applicable.
